# Short- and long-term exposure to high glucose induces unique transcriptional changes in osteoblasts *in vitro*

**DOI:** 10.1242/bio.060239

**Published:** 2024-05-14

**Authors:** Niki Jalava, Milja Arponen, Nicko Widjaja, Terhi J. Heino, Kaisa K. Ivaska

**Affiliations:** Institute of Biomedicine, Faculty of Medicine, University of Turku, Turku 20520, Finland

**Keywords:** Mesenchymal stromal cell, Osteoblast, Diabetes, High glucose, Hyperglycemia, RNA-sequencing

## Abstract

Bone is increasingly recognized as a target for diabetic complications. In order to evaluate the direct effects of high glucose on bone, we investigated the global transcriptional changes induced by hyperglycemia in osteoblasts *in vitro*. Rat bone marrow-derived mesenchymal stromal cells were differentiated into osteoblasts for 10 days, and prior to analysis, they were exposed to hyperglycemia (25 mM) for the short-term (1 or 3 days) or long-term (10 days). Genes and pathways regulated by hyperglycemia were identified using mRNA sequencing and verified with qPCR. Genes upregulated by 1-day hyperglycemia were, for example, related to extracellular matrix organization, collagen synthesis and bone formation. This stimulatory effect was attenuated by 3 days. Long-term exposure impaired osteoblast viability, and downregulated, for example, extracellular matrix organization and lysosomal pathways, and increased intracellular oxidative stress. Interestingly, transcriptional changes by different exposure times were mostly unique and only 89 common genes responding to glucose were identified. In conclusion, short-term hyperglycemia had a stimulatory effect on osteoblasts and bone formation, whereas long-term hyperglycemia had a negative effect on intracellular redox balance, osteoblast viability and function.

## Teaser

**Summary:** To understand the effect of high glucose on bone, we evaluated global changes in osteoblast transcriptome upon short- or long-term hyperglycemia and observed unique cellular responses depending on exposure time.

## INTRODUCTION

Type 2 diabetes mellitus (T2DM) is a worldwide health problem, and its prevalence is steadily growing due to our aging population and sedentary lifestyle (Chen et al., 2011; International Diabetes Federation., 2021). In addition to renal and cardiovascular complications, bone is increasingly recognized as a target for diabetic complications. T2DM patients have a lower bone turnover rate (Hygum et al., 2017; Vavanikunnel et al., 2022), an elevated risk for fragility fractures (Ferrari et al., 2018; Valderrábano and Linares, 2018), as well as delayed fracture healing (Shibuya et al., 2013) and altered bone quality (Farr and Khosla, 2016). This suggests T2DM may directly or indirectly affect bone cell function and metabolism.

T2DM is clinically characterized by elevated blood glucose level, i.e. hyperglycemia, and insulin resistance in the peripheral tissues. According to International Diabetes Federation, fasting plasma glucose level of ≤6 mM is considered healthy. Normally, postprandial plasma glucose levels will transiently raise above this level but return to normal range within 2 h (International Diabetes Federation., 2021). In patients with T2DM, postprandial plasma glucose levels may remain high for extended periods of time (Bartoli et al., 2011).

The effect of hyperglycemia on bone-forming osteoblasts and their precursors, i.e. bone marrow mesenchymal stromal cells (BMSCs), has been widely investigated. Long-term exposure to high glucose levels of ≥20 mM for more than 7 days has consistently been associated with decreased proliferation (Dong et al., 2017), mineralization and differentiation of MC3T3-E1 osteoblast-like cell line *in vitro* (Dong et al., 2017; Medeiros and Wallace, 2022; Takeno et al., 2021; Zheng et al., 2022). Decrease in proliferation (Shahen et al., 2023) and osteogenesis (Dienelt and Zur Nieden, 2011; Figeac et al., 2022; Huang et al., 2019; Zhen et al., 2010) has also been reported in cultured primary human and rodent osteoblasts. Although long-term exposure to hyperglycemia appears to impair osteoblast function *in vitro*, the molecular mechanisms are however not fully understood. High glucose is known to promote oxidative stress and accumulation of reactive oxygen species (ROS) in various cell types which has also been suggested as a potential mechanism to increase cellular senescence in BMSCs, leading to reduced proliferation and differentiation capacity (Aswamenakul et al., 2020; Tencerova et al., 2019). Additionally, high glucose has been reported to suppress the Wnt-signalling pathway in both cell lines and primary cells, resulting in suppressed osteogenesis (Kang et al., 2015; Lõpez-Herradõn et al., 2013). Osteoblasts express several glucose transporters which are required for glucose sensing and uptake (Arponen et al., 2022). However, the global transcriptomic changes modulated by hyperglycemia in osteoblasts have remained largely unexplored.

Furthermore, much less is known about the immediate effects (≤24 h) of hyperglycemia on mature osteoblasts. Impaired proliferation and motility in MC3T3-E1 cells upon 24 h exposure to 11.1 mM glucose (Pahwa et al., 2020) and reduced differentiation upon 48 h exposure to 30 mM glucose (Botolin and McCabe, 2006) have been reported. In contrast, Miranda et al. reported no changes in gene expression of *Runx2* or *Osterix* in primary human osteoblasts treated with 25 mM glucose for 24 h (Miranda et al., 2016). Differences in study design, such as cell type, glucose concentration and exposure time may explain some of the inconsistencies between various studies. Therefore, further studies to clarify the effects of short-term hyperglycemia on osteoblasts are clearly needed.

The aim of this study was to investigate the global transcriptional changes induced by high glucose environment in primary osteoblasts *in vitro*. We used rat BMSC-derived osteoblasts and exposed them to a short- (1 or 3 days) or a long-term (10 days) high glucose environment, after which global transcriptional changes were studied using mRNA sequencing. Short-term hyperglycemia was exerted on differentiated mature osteoblasts, while long-term hyperglycemia was applied over the entire differentiation period from BMSCs to osteoblasts.

## RESULTS

### BMSCs efficiently differentiated into osteoblasts *in vitro*

**Fig. 1.**
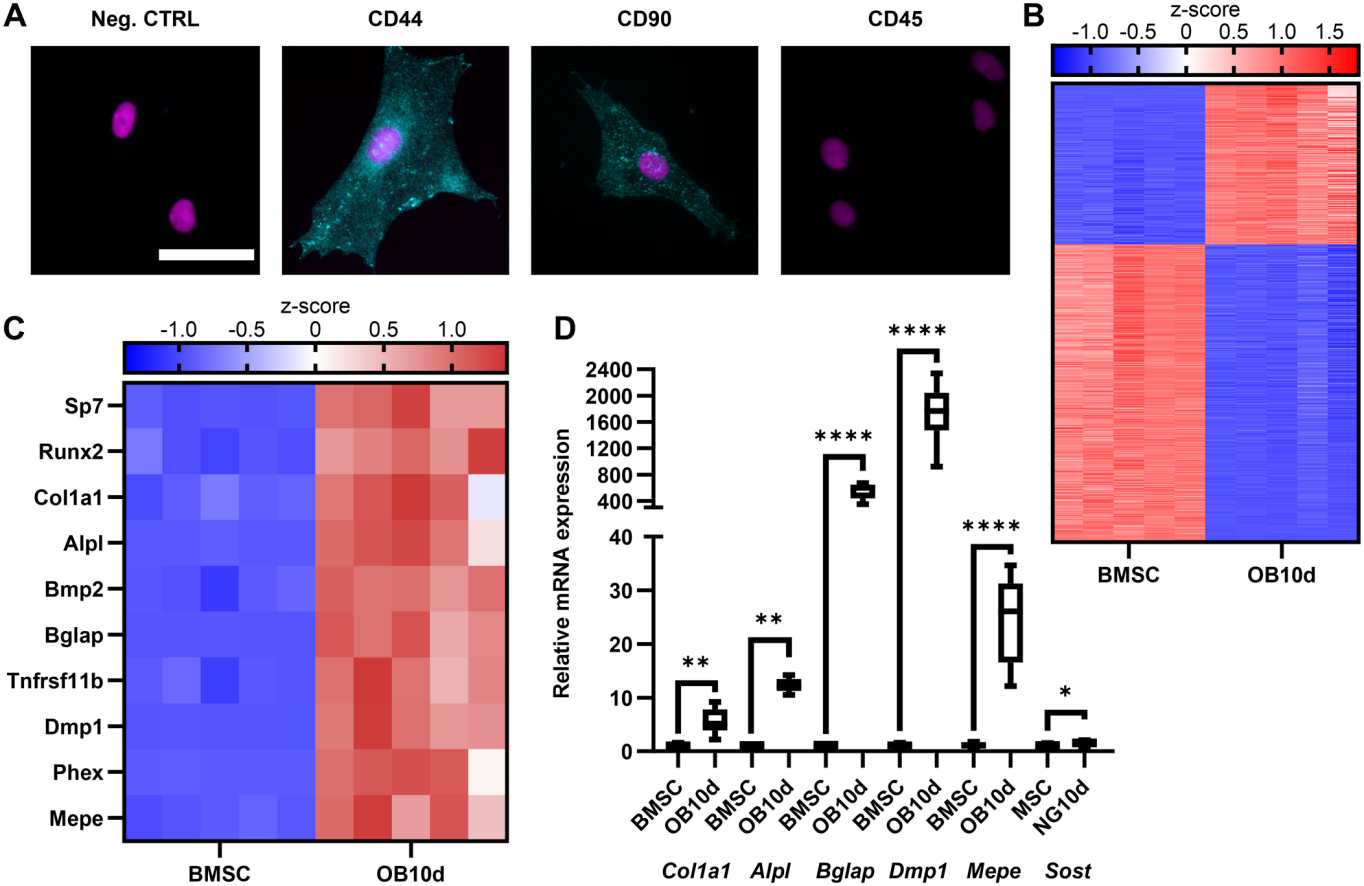
**Characterization of experimental model.** (A) rat bone marrow-derived BMSCs were stained for mesenchymal (CD44 and CD90) and hematopoietic (CD45) cell surface markers. Cell surface proteins are shown in turquoise and nucleus (DAPI) in magenta. Scale bar: 50 µm. (B) BMSCs were differentiated to osteoblasts for 10 days (OB10d) and gene expression was analyzed by RNA sequencing. Top 1000 genes with most significant adjusted *P*-value were selected for the heatmap, sorted based on fold-change from highest to lowest, and z-transformed counts are shown for each sample. Transcriptome was highly different between BMSCs and differentiated osteoblasts (*n*=5). (C) Upregulation of ten selected, osteoblast-specific genes after 10 days of differentiation. (D) Upregulation of *Col1a1*, *Alpl*, *Bglap*, *Dmp1*, *Mepe*, and *Sost*, were also confirmed by qPCR (*n*=6). *Ppib* was used as housekeeping gene to normalize mRNA levels. Student's *t*-test was used to assess statistical differences. Box-and-whiskers plots represents min-to-max distribution. Statistical significances are shown as ****P<0.0001.

We first validated our cell culture model and confirmed that the isolated BMSCs differentiate into functional osteoblasts within 10 days *in vitro*. We expanded the plastic adherent cells from initial bone marrow cell population for a total of 7 days before using them in the experiments. We confirmed BMSC-enrichment by immunohistochemical staining for mesenchymal surface markers CD44 and CD90, which were positive, and for a hematopoietic marker CD45, which was negative (Fig. 1A). Next, we verified osteoblast differentiation capability with bulk mRNA sequencing (RNA-seq). The differentially expressed genes (DEGs) were identified using DESeq2 R-package software and Benjamini-Hochberg adjustment for multiple comparison. The transcriptome analysis indeed revealed major differences between enriched BMSCs cultured for 24 h without osteogenic induction and cells differentiated into osteoblasts for 10 days, identifying 11 013 DEGs (Fig. 1B, *n*=5 replicates). Further examination of selected osteoblast-specific transcripts confirmed the upregulation of essential differentiation transcription factors *Runx2* and *Sp7/Osterix* (Fig. 1C), as well as upregulation of genes related to osteoblast function, *Col1a1*, *Alpl*, *Bglap*, *Dmp1*, and *Mepe*, which were also confirmed by qPCR (Fig. 1D), thus verifying successful osteoblast differentiation and maturity on day 10 of differentiation. A small but statistically significant increase in sclerostin (*Sost*) expression was detected with qPCR (Fig. 1D) indicating that some of the osteoblasts might have differentiated to a late-osteoblast early-osteocyte stage.

### Hyperglycemia impaired osteoblast viability

**Fig. 2.**
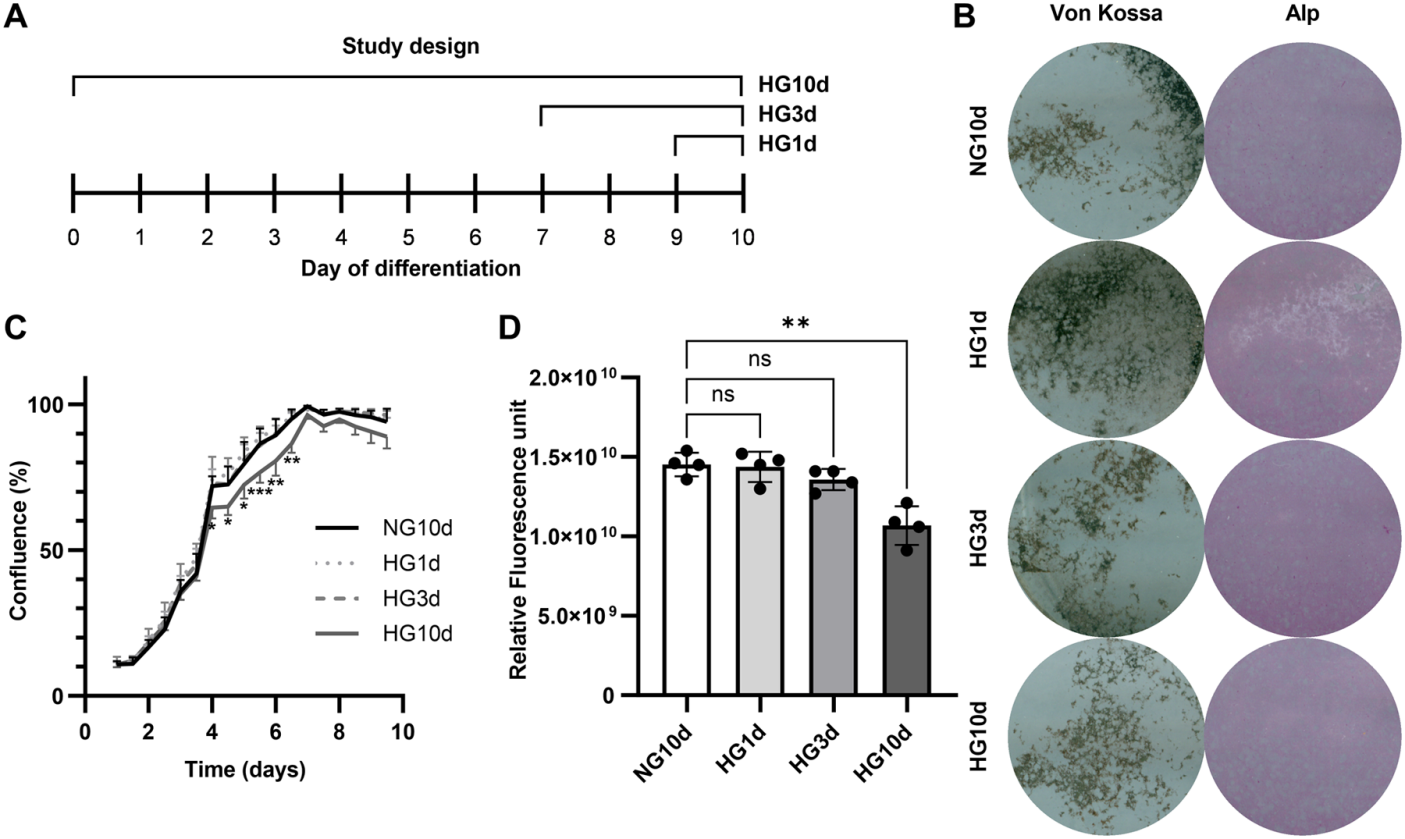
**The effects of high glucose on osteoblast growth, viability, and mineralization.** (A) Schematic presentation of the study design. (B) The effect of hyperglycemia on proliferation of BMSC-derived differentiating osteoblasts was studied over the entire duration of differentiation by measuring cell confluence with the IncuCyte Real-Time Imaging System every 12 h (*n*=4). Starting confluence for the hyperglycemic groups is normalized to NG10. (C) The effect of hyperglycemia on osteoblast viability was assessed with Calcein AM staining at the endpoint (day 10) (*n*=4). (D) The effect of hyperglycemia on mineralization and differentiation was evaluated with Von Kossa and ALP staining on day ten. Statistical differences for B and C were calculated by two-way ANOVA using Dunnet's test for multiple comparison using NG10d as the control. All experiments were repeated independently for three times. Error bars represent standard deviation. Statistical significances are shown as *P<0.05, **P<0.001.

Next, we examined the effects of short- and long-term hyperglycemia on osteoblast viability, proliferation, and function. We differentiated BMSCs into osteoblasts for 10 days. Mature osteoblasts were exposed to short-term hyperglycemia (HG, 25 mM glucose) starting on day nine for 1-day HG (HG1d) or on day seven for 3-day HG (HG10d). Long-term HG was applied on BMSCs at the beginning of differentiation for 10-day HG (HG10d). All sample collections and measurements were done on day 10. Normoglycemic culture medium (5.5 mM glucose) was used as a control (NG10d). The study design is summarized in Fig. 2A. Long-term HG exposure (HG10d) significantly impaired osteoblast proliferation (*P*<0.001, Fig. 2B), and cells reached 100% confluence later than the cells grown in NG conditions. In addition, 10 days of hyperglycemia significantly decreased the cell viability by 38% +/-16% (*P*=0.0014), as assessed by calcein staining (Fig. 2C), while 1 day (HG1d) or 3 days (HG3d) of hyperglycemia did not have significant effects on osteoblast confluence or viability (Fig. 2B,C). Metabolic viability assessed with Alamar Blue, a reagent that becomes fluorescent after reduction in the mitochondria, was decreased by 9% (*P*=0.0014) in short-term HG1d but not affected by long-term HG3d or HG10d (Fig. S1). HG10d appeared to decrease mineralization but not differentiation as evaluated by Von Kossa and alkaline phosphatase stainings, respectively (Fig. 2D). In conclusion, long-term exposure to hyperglycemia impaired the number and proliferation of osteoblasts, while short-term exposure had a modest, yet negative effect on metabolic viability.

### Short- and long-term hyperglycemia induced unique transcriptional changes on osteoblasts

**Fig. 3.**
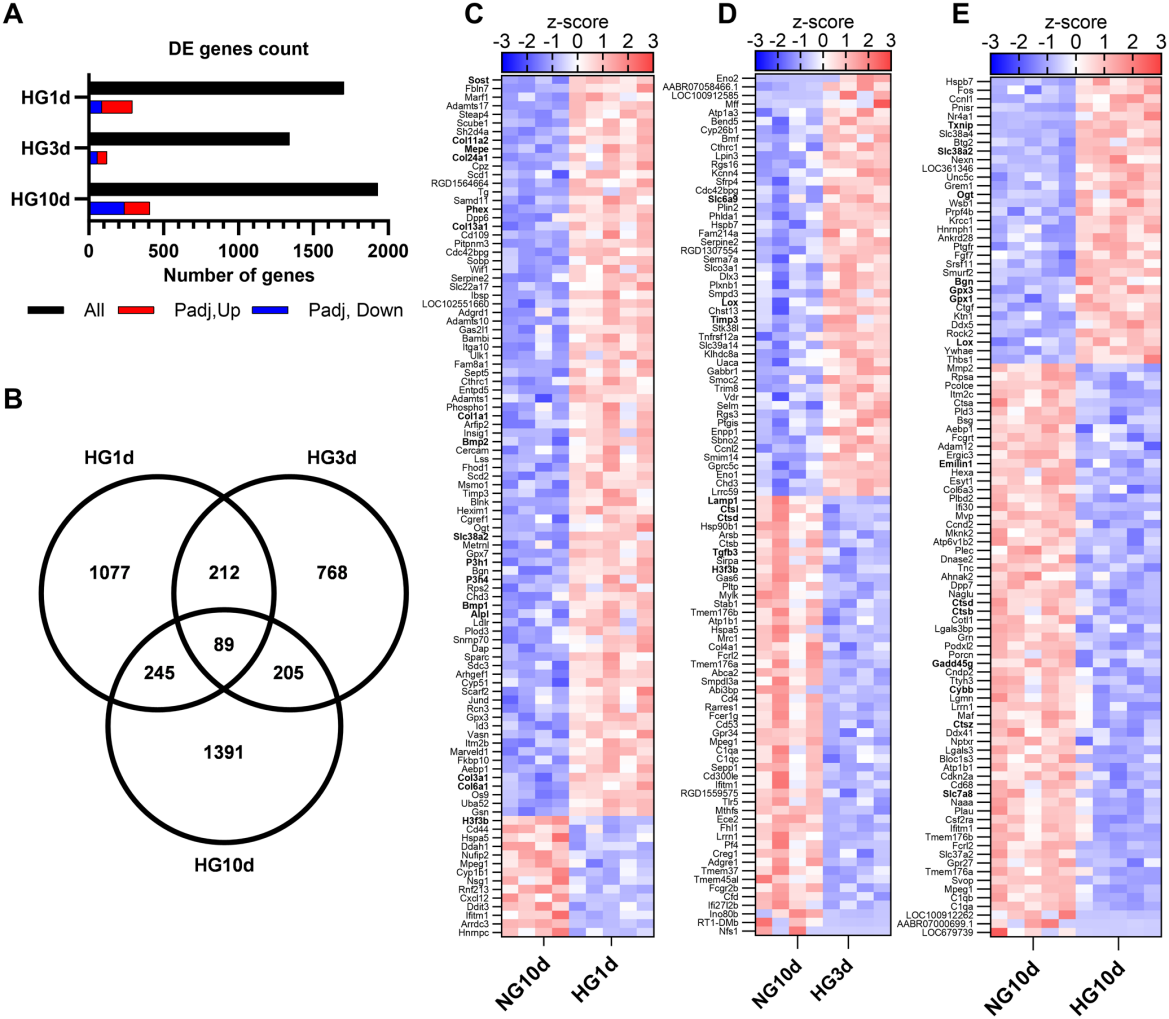
**RNA sequencing results and transcriptional changes induced by high glucose.** BMSC-derived differentiated osteoblasts were exposed to high glucose for 1, 3, of 10 days, after which the transcriptome was analyzed with RNA sequencing. (A) Number of differentially expressed genes (DEGs) per treatment before adjustment for multiple comparison, and number of up- (in red) and downregulated (in blue) genes after adjustment for multiple comparison (Benjamini-Hochberg). (B) Venn diagram of unadjusted DEGs of each glucose exposure group as compared to their respective controls. (C-E) Heatmaps of top 100 DEGs with most significant adjusted *P*-values for HG1d treatment (C), HG3d treatment (D), and HG10d treatment (E). Z-transformed counts for the DEGs are shown for each replicate and ordered by fold change from highest to lowest. Genes discussed in the results are bolded.

To further elucidate the effect of short- and long-term exposure to hyperglycemia on osteoblasts, we analyzed the global changes on transcriptome with RNA-seq. We identified 1706 DEGs in cells treated with glucose for 1 day (HG1d, *n*=294 after adjusting), and 1342 DEGs in those treated for 3 days (HG3d, *n*=121 after adjusting), when compared to cells grown in NG (Fig. 3A). For 10-day glucose treatment (HG10d), we identified altogether 1932 DEGs when compared to cells grown in normoglycemia, of which 407 remained significant after adjustment (Fig. 3A).

Interestingly, each glucose treatment triggered mostly unique transcriptional changes, with only 89 common DEGs for all three glucose exposure periods, as presented in the Venn-diagram (Fig. 3B). Individually, there were 1077 unique DEGs for HG1d, 768 for HG3d, and 1391 for HG10d. The differences are further highlighted in the heat maps of DEGs (top 100 most significant adjusted *P*-values) for each treatment (Fig. 3C-E). Short-term glucose exposure caused upregulation in the majority of the significant DEGs in both HG1d treatment (Fig. 3C) and HG3d treatment (Fig. 3D), while most of the DEGs were downregulated by glucose in the long-term exposure experiment, HG10d (Fig. 3E). Overall, the observed changes in gene expression were moderate and the fold-changes were relatively low (median fold change 0.86 for downregulation and 1.13 for upregulation). To summarize, both HG1d and HG10d resulted in over 1000 unique DEGs, but short-term HG mostly appeared to have a stimulatory effect on gene expression, while the effect of long-term HG on gene expression was predominantly suppressive. HG3d resulted in a smaller overall number of DEGs, and no distinct stimulatory or suppressive patterns could be identified.

### One-day hyperglycemia had a stimulatory effect on osteoblasts

**Fig. 4.**
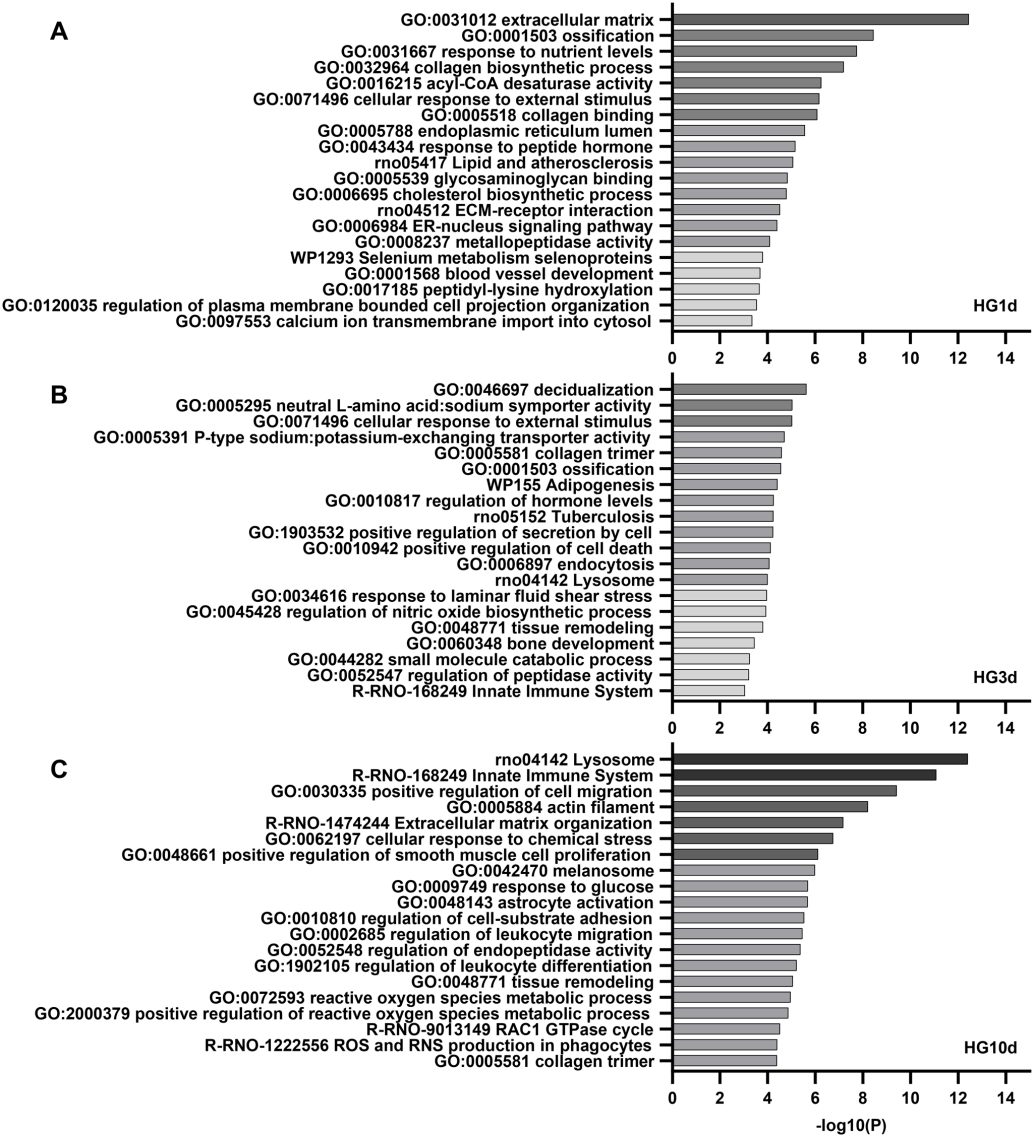
**Enriched pathway analysis based on RNA sequencing.** The top 20 enriched pathways for (A) HG1d, (B) HG3d, and (C) HG10d by analyzing the p-adjusted significant DEGs with Metascape. The pathways are sorted in descending order based on the -log_10_-transformed *P*-values. For clarification, -log_10_(*P*-value)>1.3, when *P*-value≤0.05.

**Fig. 5.**
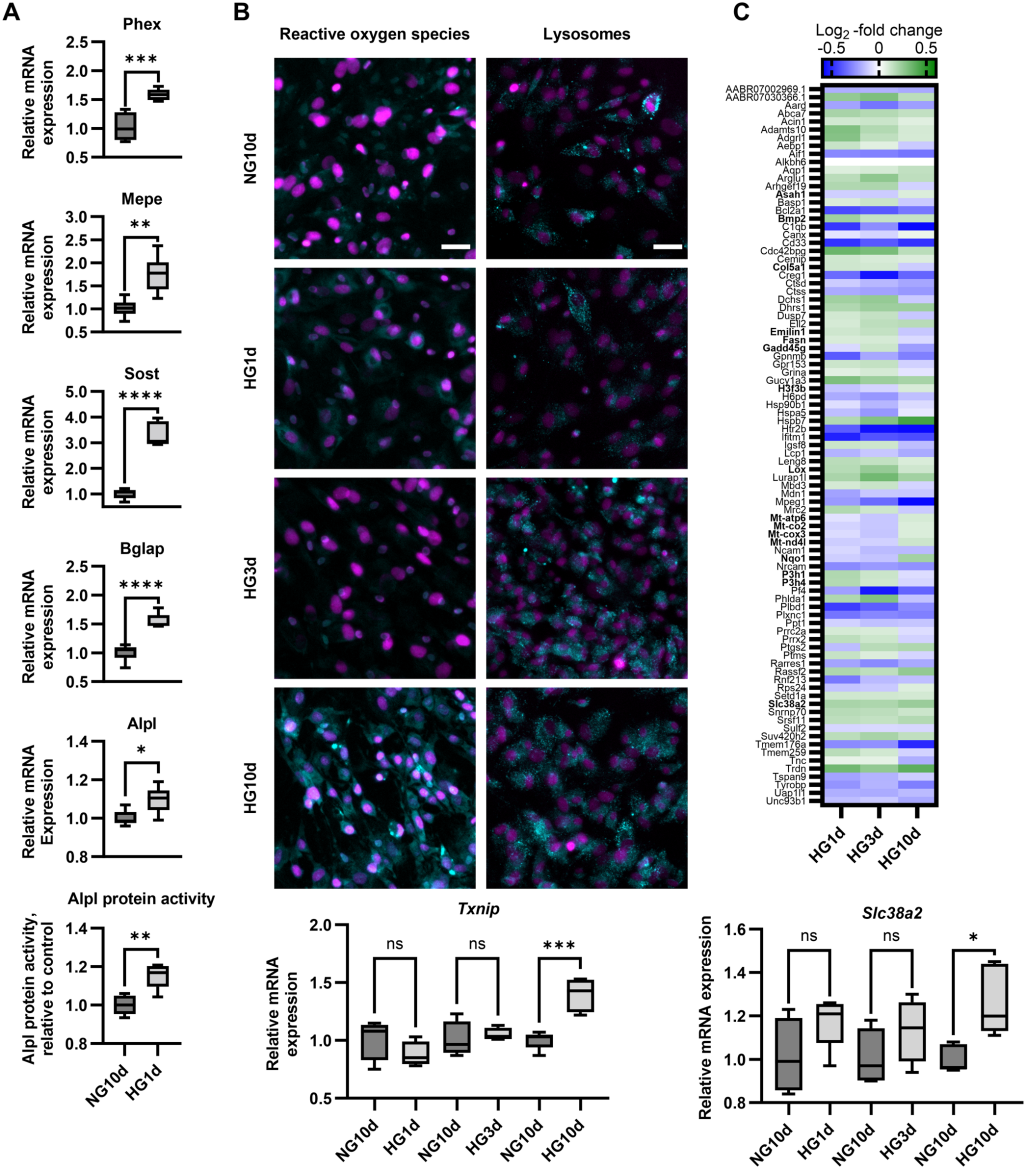
**qPCR results for selected DEGs and common changes induced by high glucose.** (A) Verification of the stimulatory effects of one-day high glucose exposure on osteogenic markers upregulated in RNA-seq. qPCR results for *Phex*, *Mepe*, *Sost*, *Bglap*, and *Alpl* expression and ALP protein activity (*n*=6). (B) The effects of high glucose exposure on reactive oxygen species (ROS). Fluorescent probes staining ROS and lysosomes (cyan) and Hoechst33258 staining the nuclei (magenta). qPCR results for *Txnip* show significantly increased expression by HG10d. Scale bars: 50 µm. (C) Common changes observed for all glucose treatment groups in the RNA sequencing. Heatmap of common DEGs (*n*=89). Genes are listed in alphabetical order and shown as log_2_ -fold change. qPCR results for *Slc38a2* show significantly increased expression by HG10d. Genes discussed in the results are bolded. The statistical differences for A were analyzed with Student's *t*-test (*n*=6) and for B and C (*n*=4-5) with one-way ANOVA using Sidak's multiple comparison adjustment to the respective NG10d control. Box-and-whiskers plots represents min-to-max distribution. All experiments were repeated independently twice. Statistical significances are shown as *P<0.05, **P<0.01, ***P<0.001, ****P<0.0001.

Pathway analysis of DEGs in HG1d glucose treatment revealed significant changes in pathways related to extracellular matrix (ECM) organization and ossification (Fig. 4A). ECM pathway included changes in the expression of several genes encoding collagens (*Col1a1*, *Col3a1*, *Col6a1*, *Col13a1*, *Col11a2*, *Col24a1*), as well as genes responsible for post-translational modifications of collagens, such as prolyl hydroxylase (*P3h1*), heat-shock protein 47 (*Serpinh1*, a collagen-specific molecular chaperone), lysyl hydroxylase 3 (*Lh3*) and lysyl oxidase (*Lox*). Within the ossification pathway, several genes important for osteoblast function, such as *Alpl*, *Mepe*, *Phex*, *Sp7/Osterix*, *Sost*, and *Bglap* were upregulated. *Phex* was the most significantly upregulated gene after 1 day glucose exposure in the dataset (1.4-fold, p_adj_=3.6×10^−28^). This upregulation was also verified by qPCR (*P*=0.0004), together with *Alpl* (*P*=0.014), *Sost* (*P*<0.0001), *Mepe* (*P*=0.0019), and *Bglap* (*P*<0.0001) (Fig. 5A). HG1d did not affect other genes encoding osteoblast-derived secreted proteins, such as *Fgf23*, *Opg*, or *Rankl* (data not shown). Despite *Sost*, a negative regulator of bone formation, was upregulated, the overall expression level of *Sost* in our experimental model was low (Fig. 1D). Taken together, 1-day treatment with high glucose upregulated genes associated with osteoblast differentiation, bone formation, collagen biosynthesis and mineralization.

### Three-day exposure to hyperglycemia had only modest effects on osteoblasts

The changes in the pathways influenced by HG3d treatment were modest and less statistically significant when compared to HG1d and HG10d treatments. Transcriptomic analysis of HG3d revealed changes in amino acid transporters, ECM interactions, and lysosome activity (Fig. 4B). For instance, several amino acid transporters (*Slc38a4*, *Slc6a9*), ECM-related genes (*Lox*, *Timp3*, *Col4a1*, *Tgfb3*), and genes involved in lysosomal activity (lysosomal-associated membrane protein 1, *Lamp1*, and cathepsins b, d, and l; *Ctsb*, *Ctsd*, *Ctsl*) were significantly downregulated upon 3-day glucose exposure. Taken together, fewer transcripts were changed when compared to 1 or 10 days of exposure, and these changes were less profound (Fig. 4B).

### Long-term hyperglycemia increased the intracellular ROS

Next, we analyzed the transcriptomic changes upon long-term glucose exposure. Enrichment analysis of DEGs revealed highly significant and unique changes in HG10d in multiple pathways, such as those related to responses to ROS and chemical stress (Fig. 4C). Increased ROS accumulation on HG10d was verified using CellROX reagent that becomes fluorescent upon oxidation (Fig. 5B), while no increase was observed with 1 or 3 days of HG. In line with this observation, genes related to ROS response, such as glutathione peroxidases (*Gpx3*, p_adj_=5.7×10^−7^; *Gpx1*, p_adj_=0.0015; Fig. 3E) and superoxide dismutase (*Sod3*, p_adj_=0.049), were upregulated by HG10d. Furthermore, thioredoxin-interacting protein (*Txnip*), which is a critical player in intracellular oxidative stress response, was the most upregulated gene by HG10d in the entire RNA-seq analysis (p_adj_=2.1×10^−26^), and its significant upregulation was also observed by qPCR (*P*=0.0002, Fig. 5B). Interestingly, *Txnip* was downregulated by HG1d treatment in RNA-seq data (p_adj_=0.016), although downregulation was not statistically significant in qPCR analysis (*P*=0.35, Fig. 5B). There was no change in *Txnip* expression by HG3d (*P*=0.94).

Additionally, long-term hyperglycemia induced significant changes in the same pathways that were observed at HG3d, such as those related to lysosomal activity and ECM organization (Fig. 4C). By utilizing LysoTracker fluorescent dye that accumulates in acidic intracellular vesicles, we observed enhanced cytoplasmic staining in both HG3d and HG10d (Fig. 5B). Although we observed enhanced staining in HG10d, all genes included in the lysosome pathway (*n*=42, *P*=3.9×10^−13^) were downregulated, including genes such as *Lamp1*, and genes encoding multiple cathepsins and other degradative enzymes (*Ctsa*, *Ctsb*, *Hexa*, *Dnase2*).

Genes included in ECM organization pathway, such as prolyl 4-hydroxylase β (*P4hb*), heat-shock protein 47 (*Serpinh1*) and matrix metalloproteinases 2 and 14 (*MMP2*, *MMP14*) were mostly downregulated by HG10d (*n*=17/20) and only three of the genes belonging to this ontology were upregulated, namely lysyl oxidase (*Lox*), hyaluronan synthase 2 (*Has2*), and biglycan (*Bgn*). Overall, long-term glucose exposure resulted in pro-oxidative intracellular environment in mature osteoblasts and reduced lysosomal activity.

### Common glucose-responsive DEGs suggested effects in proline transport and in the expression of mitochondrial genes

Finally, we evaluated in more detail those DEGs which were observed in all three glucose exposure groups, i.e. the common glucose-responsive genes in osteoblasts (*n*=89 DEGs, Fig. 5C). Responses in these genes were usually similar in direction upon both short- and long-term glucose exposures (*n*=59, of those up 24 and down 35). For example, proline transporter *Slc38a2* was one of the genes upregulated by all three glucose exposures [P_adj_=3.0×10^−6^ (HG1d), 0.044 (HG3d), and 1.05×10^−19^ (HG10d)]. Highly significant upregulation of *Slc38a2* was observed particularly in the HG10d group, which was also verified by qPCR (*P*=0.016, Fig. 5C). Despite of significant *P*-values in the RNAseq, no statistical significance was reached in the qPCR analysis for HG1d and HG3d. Interestingly, several other amino acid transporters, such as *slc1a5* (*P*=0.010), *slc7a8* (*P*=1.5×10^−5^), *slc7a5* (*P*=0.015), *slc7a15* (*P*=0.018), and *slc43a2* (*P*=0.0074) were downregulated in the RNA-seq data of HG10d. Of note, *Aldh18a1*, a gene encoding a proline precursor converting enzyme, was upregulated in HG10d (*P*=0.0008). Other genes that were upregulated by glucose in all three groups were associated with bone homeostasis (bone morphogenetic protein 2, *Bmp2*) and collagen crosslinking (lysyl oxidase, *Lox*).

Genes upregulated by short-term (HG1d and HG3d) but downregulated by long-term glucose (HG10d) (*n*=22) included several genes related to collagen formation and modifications (such as collagen 5a1, *Col5a1*; elastin microfibril interfacer 1, *Emilin1*; prolyl hydroxylases, *P3h1*, *P3h4*). Genes downregulated by short-term but upregulated by long-term glucose (*n*=8) included four mitochondrially-encoded electron transport chain genes (*Mt-co2*, *Mt-cox3*, *Mt-nd4l*, *Mt-atp6*), NAPDH dehydrogenase (*Nqo1*) and histone H3.3 (*H3f3b*), which has a role in regulation of chromatin integrity (Fig. 5C). Staining of mitochondria in osteoblasts in all glucose treatment groups did not however show any differences between the groups (Fig. S1).

## DISCUSSION

In this study, we investigated global transcriptional changes upon short- and long-term hyperglycemia (25 mM) on primary rat osteoblasts *in vitro* and found that responses were dependent on the duration of glucose exposure. Short-term exposure appeared to have a stimulatory effect on genes related to matrix mineralization and ECM production but also in the regulation of bone formation, whereas long-term hyperglycemia had a negative effect on intracellular redox balance and osteoblast viability.

One day (24 h) exposure to high glucose resulted in more upregulated than downregulated genes in our RNAseq analysis. Transcripts which were highly upregulated by short-term hyperglycemia were related to ECM production, such as the genes encoding several collagens, as well as genes responsible for post-translational modifications required for collagen fibril formation. Another clearly upregulated pathway included several genes related to bone formation and bone matrix mineralization (*Alpl*, *Bglap*, *Sost*) whose upregulation was further verified by qPCR. Some of these transcripts encode secreted proteins (Mepe, Bglap, Sost), suggesting that acute high glucose on osteoblasts could as well have indirect effects on other cells and tissues. Increased expression of Sost by high glucose has also been reported in osteocyte-like cells after long-term exposure (>28 days) (Pacicca et al., 2019). Interestingly, upregulation of ossification-related genes was not observed when glucose exposure was extended to 3 days (72 h) suggesting that the positive effects of high glucose had already been attenuated by that time. In MC3T3-E1 cells, the results for 24 h exposure to high glucose are mixed; others have reported increased collagen deposition but decreased mineralization (Cunha et al., 2014) or no effect on proliferation or mineralization (Liu et al., 2015). Increased proliferation, differentiation and collagen production has been reported in response to short-term (≤24 h) hyperglycemia in other cell types, such as cardiac fibroblasts (Shamhart et al., 2014) and increased cell adhesion and transmigration in aortic endothelial cells (Piga et al., 2007), supporting the stimulatory effects. We also noticed a small decrease in mitochondrial activity measured by Alamar Blue viability assay. There were no detectable differences in mitochondrial staining with MitoTracker, suggesting only modest and possibly transient suppressive effect on mitochondrial function after acute high glucose exposure. Altogether, our results suggest that *in vitro* short-term exposure to high glucose environment has a transient stimulatory effect on osteoblast function, matrix production and mineralization.

In contrast, 10-day exposure to high glucose seemed to have a suppressive effect on osteoblasts, with more downregulated than upregulated genes observed in RNAseq. Cell viability and mineralization were also significantly impaired. This is in line with previous studies, in which long-term hyperglycemia has been shown to have negative effects on osteoblast proliferation and bone formation, as evaluated by the expression of selected osteoblast-specific genes and mineralization (Balint et al., 2001; Liu et al., 2015; Takeno et al., 2021). However, the global transcriptional changes induced by chronic hyperglycemia have not been previously assessed on primary BMSC-derived osteoblasts. We observed that long-term hyperglycemic exposure of 10 days resulted in suppression of ECM-related pathways but also impaired the ability of osteoblasts to produce and mineralize extracellular matrix. Even though decreased osteoblast function has been attributed to increased cellular senescence (Tencerova et al., 2019), we did not observe statistically significant changes in senescent markers at transcriptional level (data not shown). Long-term BMSC cultures in high glucose have been shown to exhibit decreased differentiation capability without indication of senescence (Al-Qarakhli et al., 2019). Xia et al. (2022) studied BMSCs from osteoporotic patients with or without T2DM using RNA-seq and noticed that T2DM-derived BMSCs exhibit decreased differentiation and mineralization capacity, which they attributed to attenuated expression of *Forkhead Box Q1* (FOXQ1). However, we did not observe changes in FOXQ1 in our dataset.

One possible mechanism for the negative effects observed by long-term hyperglycemia is impaired intracellular redox homeostasis. Hyperglycemia and mitochondrial dysfunction increase the accumulation of intracellular ROS, which contribute to inflammation and impairment of various cellular processes causing DNA damage, cellular senescence, and apoptosis (Kang et al., 2015; Morgan and Liu, 2010; Rharass and Lucas, 2019). Accumulation of ROS may be one of the mechanisms responsible for cellular defects and complications related to diabetes, such as retinopathy (Chan et al., 2020) and impaired wound healing (Buranasin et al., 2023). Interestingly, increased intracellular ROS production in response to high-glucose environment has been reported to occur also in bone cells (Zeng et al., 2021; Zhen et al., 2010), suggesting that ROS might contribute to bone-related complications of T2DM. In our study, 10 days of hyperglycemia increased the expression of genes within ROS-related ontology pathways and increased oxidative stress, suggesting that long-term hyperglycemia increases ROS accumulation, intracellular stress, and damage in osteoblasts. The imbalance of oxidative stress is further supported by the upregulation of *Txnip*, which was the most significantly upregulated gene by long-term hyperglycemia in our dataset. *Txnip* has previously been shown to be strongly induced by hyperglycemia in many other cell types, such as pancreatic beta cells, retinal epithelial cells and human MSCs (Chen et al., 2008; Devi et al., 2019; Jiang et al., 2022). TXNIP acts as pro-oxidative, pro-inflammatory and pro-apoptotic protein. It suppresses the activity of thioredoxin system which is required for the removal of intracellular ROS and for protecting cells from oxidative damage (Devi et al., 2019; Li et al., 2007). Upregulation of *Txnip* upon long-term high glucose exposure may thus reduce the capability of osteoblasts to neutralize harmful ROS and to withstand hyperglycemia-related cellular toxicity, and further studies are warranted to elucidate the role of *Txnip* in osteoblasts.

We also aimed to identify common glucose-responsive genes, irrespective of the duration of hyperglycemia. Interestingly, only 89 DEGs were common for all three hyperglycemia exposures, i.e. 1, 3 and 10 days of hyperglycemia. One of the genes that was upregulated by all three exposure times was *Slc38a2*. It encodes an S-type amino acid transporter known to be responsible for proline transport into the osteoblasts (Mackenzie et al., 2004; Shen et al., 2022). In addition, another S-type amino acid transporter, *Slc38a4*, was significantly upregulated by 3- and 10-day high glucose exposures. Increased need for proline is further supported by the upregulation of *Aldh18a1*, a gene encoding an enzyme which is responsible for the synthesis of proline precursor from glutamate. Osteoblasts express several proline-rich proteins, such as collagens and osteocalcin, and thus have a higher demand for proline than many other cell types (Karna et al., 2020; Shen et al., 2022). Excess availability of glucose may therefore allow osteoblasts to increase their proline uptake and collagen biosynthesis. We further observed downregulation of many other amino acid transporters such as *Slc1a5* and *Slc7a5* by long-term hyperglycemia. The lack of these two glutamine transporters has been associated with impaired differentiation of osteoblasts (Ni et al., 2021; Sharma et al., 2021; Shen et al., 2021) indicating that the glutamine/glutamate to proline ratio may be important for osteoblast differentiation and proliferation.

Exposure to hyperglycemia appeared to have an effect also on pathways related to cellular organelles, such as mitochondria and lysosomes. Transcripts encoded by mitochondrial DNA (mtDNA, in total 13 protein-encoding genes) were changed in all three datasets, but not always in the same direction. Four (out of 13) were changed by HG1d, nine by HG3d and eight by HG10d. Mitochondrial transcripts were upregulated by long-term exposure and downregulated by short-term exposures. However, we did not observe detectable differences in the abundance of mitochondria with fluorescent staining. Nevertheless, changes observed in the RNA-seq may reflect changes in the energy metabolism or in the biogenesis of mitochondria and mtDNA. In agreement with our results, hyperglycemia has been previously shown to result in dysfunctional mitochondria and defects in mitochondrial biogenesis in MC3T3-E1 osteoblasts (Pahwa et al., 2020) indicating mitochondrial defects could contribute to impaired osteoblast function. Downregulation of the lysosomal degradation pathways and the increased fluorescent staining of acidic vesicles upon both HG3d and HG10d treatments was somewhat unexpected. This may indicate decreased lysosomal degradation leading to accumulation of endogenous material in lysosomes. Nevertheless, all genes included in the lysosome ontology pathway (*n*=42) were consistently downregulated by long-term hyperglycemia. Lysosomal alterations have also been observed in diabetic skin cells by RNA-seq (Zhang et al., 2021). Of note, upregulation of *Txnip* by hyperglycemia has been associated with mitochondrial fragmentation and mitophagic flux to lysosomes in retinal pigment epithelium cells (Devi et al., 2019), suggesting a putative link between ROS generation and mitochondrial and lysosomal dysfunction. Whether this takes place also in osteoblasts, remains to be investigated.

To the best of our knowledge, this is the first study profiling the global effects of hyperglycemia on osteoblast transcriptome in primary BMSC-derived cells. Furthermore, the effects of short-term hyperglycemia on osteoblasts have not been previously reported. The strengths of our study include the well-characterized osteoblast differentiation model (Arponen et al., 2022) and three different exposure times with high glucose. To facilitate data interpretation, we used a single concentration of hyperglycemia, 25 mM, which is a commonly used model to study hyperglycemia *in vitro* (e.g. Cunha et al., 2014; Takeno et al., 2021; Zheng et al., 2022). Overall, the effects of hyperglycemia on gene expression were modest, which can be expected for a relatively physiological stimulus, such as glucose availability. However, it should be noted that local glucose concentrations in bone microenvironment *in vivo* are largely unknown. One limitation of our study is the lack of proteomic profiling which was not performed since the aim was to map global transcriptional changes. Furthermore, it should be noted that our results were obtained using primary osteoblasts isolated from Sprague Dawley rats, and thus the results cannot be directly translated into human osteoblasts. Further studies to elucidate the molecular mechanisms responsible for the harmful effects of high glucose are still warranted. It would be of great interest to study if glucose-induced changes in osteoblasts affect bone metabolism *in vivo*.

In summary, both short- and long-term exposure to high glucose environment induces unique transcriptional changes in osteoblasts. Short-term hyperglycemia has a stimulatory effect on genes and pathways related to bone matrix production and mineralization, while long-term exposure impairs osteoblast function, most likely via mechanisms involving imbalance in intracellular oxidative stress and upregulation of *Txnip*. Direct effects of high glucose on osteoblasts may explain some of negative skeletal features seen in T2DM patients. Whether more transient exposure of osteoblasts to high glucose has a positive effect on bone formation also *in vivo* remains to be elucidated.

## MATERIALS AND METHODS

### Cell culture

BMSCs were isolated from femurs and tibias of 4-week-old Sprague-Dawley rats. Animal experimentation was approved by the local review committee of Central Animal Laboratory, University of Turku (Turku, Finland). Briefly, femurs and tibias were dissected, and the bone marrow (BM) was evacuated by centrifugation. BMSCs were enriched by plastic adherence for 2 days and expanded for additional 5 days for a total of 7 days before collecting, seeding, and using them in the experiments. The expansion was done in basal medium containing α-Minimum Essential Medium [αMEM (5.5 mM glucose), Gibco, Thermo Fisher Scientific, USA] with 15% inactivated fetal bovine serum (iFBS, Gibco), HEPES (10 mM, Gibco), GlutaMAX™ (2 mM, Gibco), and Penicillin-Streptomycin (100 U/ml, 100 µg/ml, Gibco). During the expansion, the media was additionally supplemented with Amphotericin B (2.5 µg/ml, Gibco) and dexamethasone (10^−8^ M, Sigma, USA). All cell cultures were performed at 37°C and 5% CO_2_.

BMSCs were differentiated to osteoblasts for 10 days using osteogenic medium (OM) containing basal medium with 10% iFBS, supplemented with Na-β-glycerophosphate (10 mM, Fluka BioChemika, Switzerland) and L-ascorbic acid 2-phosphate (70 µg/mL Sigma, USA). BMSCs were seeded on 6-well plates at 40,000 cells per well in six replicates. Undifferentiated BMSCs were cultured for 24 h at 300,000 cell per well (*n*=6) without the osteogenic supplementation.

### Short- and long-term HG

HG was introduced by adding D-glucose to culture medium for up to 10 days. HG medium was made by adding 19.5 mM D-glucose (Sigma, USA) to the respective normoglycemic (NG) medium containing 5.5 mM glucose, resulting in the final glucose concentration of 25 mM. Glucose concentration in culture media was confirmed with GlucCell glucose monitoring system (CESCO Bioengineering Co., Ltd, Taiwan) (data not shown).

For short-term exposure, HG was introduced to osteoblasts on day 9 of differentiation, 1 day before sample collection (HG1d); or on day 7 of differentiation, 3 days prior to sample collection (HG3d). For long-term exposure, HG was introduced on day 0 for the entire duration of differentiation (10 days, HG10d). Culture medium was changed completely on days 4 and 7, and additionally on day 9 for the short-term exposure. Osteoblasts grown in NG media served as controls.

### RNA isolation and qPCR

Cell lysates were collected at indicated time points and RNA was isolated using Macherey-Nagel Nucleospin RNA Plus kit according to the manufacturer's instructions with the following modifications. The samples were collected into the lysis buffer using a cell scraper and stored at −85°C until isolation. After thawing, RNA lysates collected at day 10 were additionally homogenized with a homogenizer (UltraTurrax T25, Janke and Kunkel IKA-Labortechnik, Germany) in three 1-3 s bursts at 20,000 rpm prior to RNA isolation to ensure the breakdown of ECM. RNA was eluted in RNase-free water and RNA concentrations were measured with spectrophotometer NanoDrop One (Thermo Fisher Scientific, USA). Isolated RNAs were stored at −85°C.

For qPCR analysis, 1 µg of RNA was reverse transcribed to cDNA with Maxima Reverse Transcriptase kit (Thermo Fisher Scientific, USA), according to the manufacturer's instructions. qPCR analysis was done on the Bio-Rad CFX96 thermal cycler (Bio-Rad Laboratories, USA) system using Dynamo SYBR Green HS (Thermo  Fisher Scientific, USA). The following genes were analyzed with primers purchased from Oligomer (Finland) or Integrated DNA Technologies (Belgium): alkaline phosphatase (*Alpl*), osteocalcin (*Bglap*), matrix extracellular phosphoprotein (*Mepe*), osteoprotegerin (*Opg*), X-linked phosphodiesterase (*Phex*), cyclophilin B (*Ppib*), receptor activator of nuclear factor κ-B ligand (*Rankl*), Runt-related transcription factor 2 (*Runx2*), solute carrier 38a2 (*Slc38a2*), sclerostin (*Sost*), and thiredoxin-interacting protein (*Txnip*). Primers are listed in Table S1. Data were analyzed by ΔΔCT-method (Livak and Schmittgen, 2001) and mRNA expression was normalized to cyclophilin B expression and is presented relative to undifferentiated or untreated samples, as indicated.

### Analysis of gene expression by RNA sequencing

mRNA sequencing (RNA-seq) technology was utilized to analyze global changes to the transcriptome upon short and long-term HG treatments with respective NG controls (*n*=5-6/ group).

mRNA library preparation, RNA-seq, and bioinformatic analysis was purchased from Novogene, Ltd. (Cambridge, UK). In brief, RNA quality and integrity was first evaluated with BioAnalyser Agilent 2300 system. mRNA library suitable for RNA-seq was prepared via poly-A enrichment. Samples were then sequenced using Illumina NovaSeq platform (Illumina NovaSeq 6000 sequencing system) using paired-end 150 bp strategy and 20 M reads per sample.

The raw reads were subjected to quality control in which the error rate, GC content, and read quality were evaluated and consequently bad quality reads were filtered. For the read alignment, HISAT2 algorithm was used to map the reads to *Rattus norvegicus* reference genome (Rnor_6.0). Alignment data were used for calculation of gene expression levels based on read counts. Differential expression analysis was performed using the DESeq2 R-package (Anders and Huber, 2010) and the resulting *P*-values were adjusted for false discovery rate using the Benjamini-Hochberg multiple testing adjustment procedure (Benjamini and Hochberg, 1995). Functional analysis of the DEGs was done by Metascape online tool (Zhou et al., 2019) using minimum of five genes overlap and 3.0 enrichment score. In the bioinformatic analyses adjusted *P*-values <0.05 were considered statistically significant.

In the short-term transcriptome analysis (*n*=5), we identified one sample in both NG10d control groups and one in the HG3d that were clustering clearly differently when compared to their biological replicates (Fig. S2A). We excluded those three outlier samples from further analysis, resulting in four replicates for control groups NG10d (control for HG1d) and NG10d (control for HG3d) as well as for the HG3d group. In the long-term transcriptome analysis (*n*=6), we further identified one sample in both NG10d and HG10d groups that clustered differently from the other biological replicates. We excluded those samples resulting in five replicates in both groups (Fig. S2B).

### Cell viability

The cells were grown on a 24-well plate at 10,000 cells per well as four replicates. Temporal changes in cell confluence during the 10-day culture period were assessed in the short- and long-term high glucose environment with IncuCyte S3 Live-Cell Analysis Instrument (Sartorius), capturing phase contrast micrographs at 12 h intervals. On day 10, wells were washed with PBS and 1 mM Calcein AM (Life Technologies, USA) solution was added for 45 min (+37°C) after which fluorescence intensity and confluency were measured with IncuCyte S3 software. Viability and confluency were assessed with user-defined settings. Metabolic viability was assessed with Alamar Blue HS Cell Viability Reagent (Invitrogen, USA) according to the manufacturer's instructions. Fluorescence was measured in duplicates with excitation/emission set at 560/590 nm (Ensight Multimode Plate reader, Perkin Elmer, USA).

### Cytochemical staining

Cells were fixed with 4% PFA for 20 min, and mineralization was assessed with von Kossa staining on day 10. In brief, the staining was done by first washing the wells with H_2_O and then incubating the samples with 2% AgNO_3_ for 1 h at room temperature (RT) under a 20 W table lamp. After washing, 2.5% Na_2_S_2_O_3_ was added and incubated for 5 min, washed and air-dried. Alkaline phosphatase (ALP) was stained on day ten using Alkaline Phosphatase Leukocyte Kit (Sigma) according to the manufacturer's instructions. The stained wells were imaged using HP ScanJet G4010 (Hewlett-Packard Company, USA).

Oxidative stress was assessed using CellROX Green (Life Tech, USA) and the mitochondria were stained using MitoTracker Orange CMTMRos (Molecular Probe, USA) fluorochrome probes according to the manufacturers' protocols, respectively. In brief, 5 µM CellROX Green or 250 nM MitoTracker Orange was prepared in serum-free medium (αMEM, Gibco, Thermo Fisher Scientific, Inc, USA) and prewarmed to 37°C. The staining solutions were then applied on cells and incubated at 37°C, 5% CO_2_ for 30 min, after which the cells were fixed with 4% PFA at RT for 10 min while protected from light. The nuclei were stained with 5 µg/ml Hoechst 33258 in PBS for 5 min. Acidic vesicles were stained using LysoTracker Red DND-99 (Molecular Probes, USA) fluorochrome dye. 50 nM LysoTracker Red was used concurrently with 5 µg/ml Hoechst 33258 to stain also the nuclei, and the solution was prepared to serum-free medium (αMEM, Gibco) and prewarmed to 37°C. Staining solution was then applied to cells and incubated at 37°C, 5% CO_2_ for 30 min. The cells were imaged immediately after fluorochrome stainings using EVOS fluorescent cell imaging system (Thermo Fisher Scientific, Germany) under standardized imaging setup. The background noise was subtracted using cell-free but fluorochrome-stained control wells in ImageJ 1.53c-software and pseudo-colored cyan (CellROX, MitoTracker, LysoTracker) and magenta (Hoechst 33258).

### Alkaline phosphatase activity

ALP enzymatic activity was measured from cell lysates on day ten. The cells were lysed in 50 mM Tris-HCl, 0.1% Triton X-100, 0.9% NaCl, pH 7.6, after which they underwent three freeze-thaw cycles before assessing the enzymatic activity. The lysates were incubated with 0.15 nM PNPP (0.1 M Tris, 1 mM MgCl_2_, pH 10) as a substrate for 45 min, after which the reaction was stopped using 1 M NaOH. Absorbance was measured at 405 nm (Ensight Multimode Plate reader, Perkin Elmer, USA) and normalized to total protein measured using Bradford protein assay (Bio-Rad Laboratories).

### Immunohistochemical staining

BMSCs were seeded on a glass coverslip and let to attach at 37°C, 5% CO_2_ for 24 h. The cells were fixed with 4% PFA at RT for 15 min, permeabilized with 0.05% Triton X-100 at RT for 5 min and blocked with 10% normal goat serum (Abcam, UK) at RT for 1 h. Primary antibodies were added and incubated at +4°C overnight. The following antibodies and concentrations were used: rabbit anti-CD44 (Abcam, UK; 0.2 μg/ml, ab157107), rabbit anti-CD45 (Merck, USA; SAB4502541, 10μg/ml), and mouse anti-CD90 (Abcam, UK; ab225, 1 μg/ml). Cells were then washed three times with 0.1% Tween-20 and incubated at RT for 1 h with the following secondary antibodies: Alexa Fluor^®^ 488-conjugated goat anti-rabbit IgG (Abcam, UK; ab150077, 2 μg/ml) and Alexa Fluor^®^ 594-conjugated goat anti-mouse IgG (Abcam, UK; ab150116, 2 μg/ml). Nuclei were stained with Vectashield Antifade with 1.5 µg/ml DAPI (Vector Laboratories, USA). Stained cells were then imaged with Zeiss Axio Imager 1 (Zeiss, Germany) under standardized imaging setup. Images were processed in Zen Blue 2 (Zeiss) and ImageJ 1.53c software and pseudo-colored cyan (secondary antibodies) and magenta (DAPI).

### Statistical analysis

All data is presented as mean values with standard deviations. Data were first tested for normal distribution and equal variance with Shapiro-Wilk and Levene's tests, respectively. For normally distributed parameters, parametric test was chosen, otherwise an equivalent non-parametric test was chosen. For qPCR results, one-way analysis of variance (ANOVA) was used when comparing multiple groups with Sidak's adjustment for multiple comparison, and Student's *t*-test was used when multiple comparison was not required. For viability and temporal growth assessment, two-way ANOVA was used with Dunnet's adjustment for multiple comparison using NG10d as the control. Each specific test is also mentioned in the respective figure legend. The number of replicates in each cell culture experiment was 4-6, as indicated in each figure legend. All cell culture experiments were independently replicated for the minimum of two times using freshly isolated BMSCs for each experiment. GraphPad Prism 9 software were used for statistical analyzes. *P*-values <0.05 were considered statistically significant.

## Supplementary Material

10.1242/biolopen.060239_sup1 Supplementary information
