# Supplementary material for: Short- and long-term exposure to high glucose induces unique transcriptional changes in osteoblasts in vitro
Source: Biol Open. 2024 May 14;13(5):bio060239. doi: 10.1242/bio.060239 (PMC11128269; doi:10.1242/bio.060239)
Supplement: Supplementary information [file biolopen-13-060239-s1.pdf]

Metabolic viability and mitochondrial staining

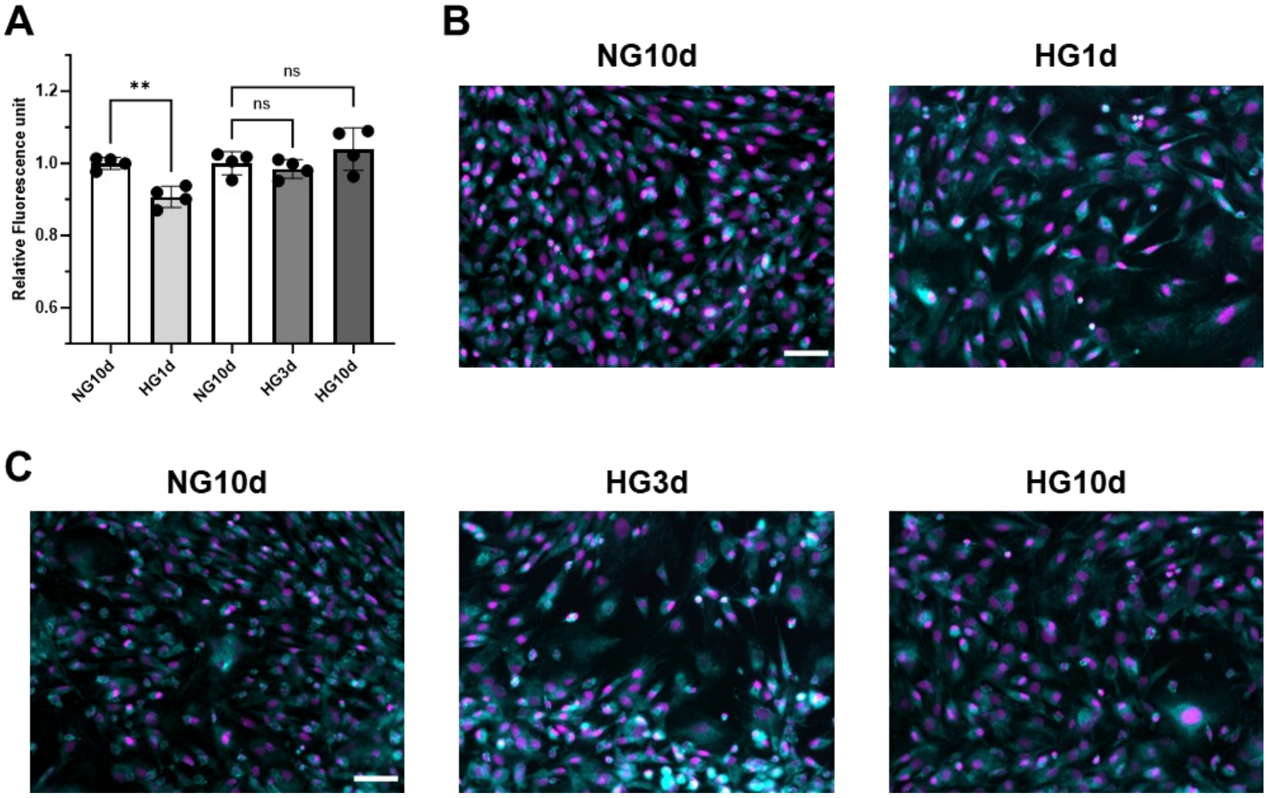

Fig. S1. Metabolic viability and mitochondria staining images. A) Metabolic viability of BMSC-derived differentiated osteoblasts assessed with AlamarBlue reagent. Metabolic viability was decreased 9% after one-day high glucose exposure but not after three- or ten-day exposure. Mitochondria were stained with MitoTracker fluorescent probes after the cells were exposed to high glucose for B) one or C)three and ten days. MitoTracker staining the mitochondria (cyan) and Hoechst 33258 staining the nuclei(magenta). Exposure to high glucose did not have a noticeable effect on mitochondria. Scale bars 100 µm. Statistical significance for A) was tested with ANOVA using Sidak's adjustment method to correct for multiple testing.

Biology Open • Supplementary information

Clustering heatmaps for all groups

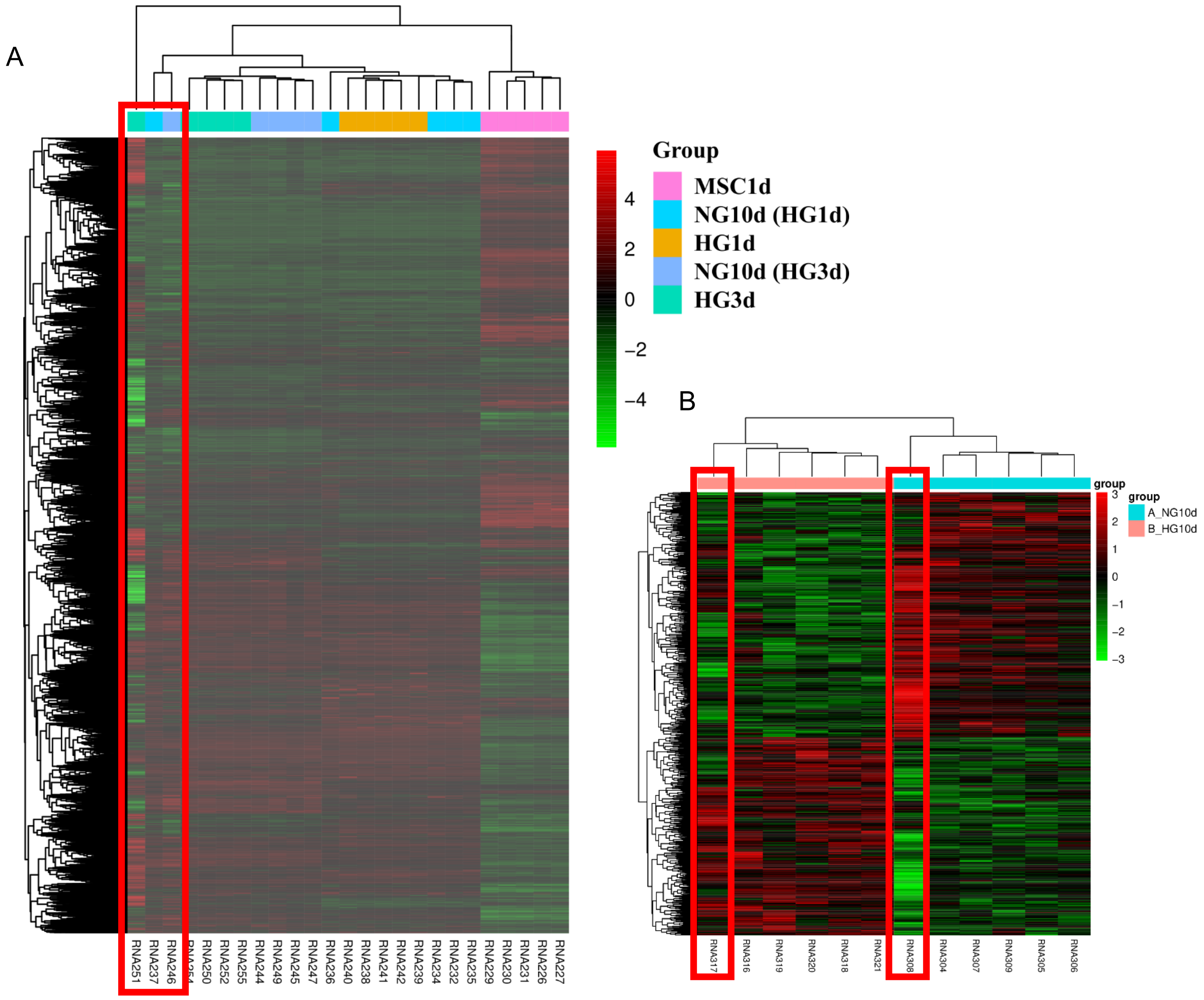

Fig. S2. Heatmap clustering of gene expression across all samples was analysed to validate sample quality, A) RNAseq for MSCs, HG1d and HG3d experiment. Clustering revealed that RNA246 (NG10d, control for HG3d), RNA251 (NG10d, control for HG1d) and RNA237 (HG3d) were different from other biological replicates and clustered separately (highlighted by red box). **B)** RNAseq for HG10d experiment. RNA308 (NG10d) and RNA317 (HG10d) were different from other five biological replicates and clustered separately (highlighted by red box). Based on clustering profile, we excluded samples RNA237, RNA 246, RNA251, RNA308, and RNA317 from all the analysis.

Biology Open • Supplementary information

Primer sequence table

Table S1. Primer sequences (in alphabetical order).

Gene	NCBI code	Sequence	Annealing temperature
Alpl	NM_013059.1	F: 5'-TGCAGGATCGGAACGTCAAT-3'<br>R: 5'-GGGTCTTTCTCTTTCTCTGGCA-3'	60°C
Bglap	NM_013414.1	F: 5'-TGAGTCTGACAAAGCCTTCATGT-3'<br>R: 5'-GAAGCCAATGTGGTCCGCTA-3'	61°C
Col1a1	NM_053304.1	F: 5'-CACTGCAAGAACAGCGTAGC-3'<br>R: 5'-AGTTCCGGTGTGACTCGTG-3'	59°C
Dmp1	NM_203493.4	F: 5'-GTCCTGTGCTCTCCCTGTCG-3'<br>R: 5'-CACTGCTGTCCGTGTGGTCA-3'	61°C
Fgf23	NM_130754.1	F: 5'-AGGATGCTGGCTCCGTAGTG-3'<br>R: 5'-CGGGCTGAAGTGATACGATCCA-3'	62°C
Mepe	NM_024142.1	F: 5'-GAAGGTGAACGACACCAGAGAG-3'<br>R: 5'-GGCTCAGGCTTCACAGATGC-3'	59°C
Opg	NM_012870.2	F: 5'-AGACGTCATCGAAAGCACCC-3'<br>R: 5'-GCACAGGGTGACATCTATTCCA-3'	58°C
Phex	NM_013004.2	F: 5'-GGGAACAGAATACCCTCGATCTC-3'<br>R: 5'-CACAGACCACCACGGATCAA-3'	62°C
Ppib	NM_022536.1	F: 5'-ACCTGTAGGACGAGTGACCT-3'<br>R: 5'-GCTCTTTCCTCCTGTGCCAT-3'	60°C
Rankl	NM_057149.1	R: 5'-GCTCTTTCCTCCTGTGCCAT-3'<br>R: 5'-AAGATAGTCCGCAGGTACGC-3'	58°C
Runx2	NM_053470.2	F: 5'-CGCCTCACAAACAACCACAG-3'<br>R: 5'-TGCAGCCTTAAATATTACTGCATGG-3'	60°C
Slc38a2	NM_181090.3	F: 5'-CATCTTCGGGTTCATTGGTGC-3'<br>R: 5'-CATCACGACTACGCCACTCA-3'	60°C
Sost	NM_030584.2	F: 5'-GCCTTCGTTGCTGTGGAGAG-3'<br>R: 5'-TGTACTCGGACACGTCTTTGGT-3'	61°C
Txnip	NM_001008767.2	F: 5'-CCATTCTGGGCTGCAACATC-3'<br>R: 5'-TATAGCAAGGTGGAGCTTCTGG-3'	61°C
